# Cognitive Outcomes in Early-Treated Adults With Phenylketonuria (PKU): A Comprehensive Picture Across Domains

**DOI:** 10.1037/neu0000337

**Published:** 2017-01-12

**Authors:** Liana Palermo, Tarekegn Geberhiwot, Anita MacDonald, Ellie Limback, S. Kate Hall, Cristina Romani

**Affiliations:** 1 School of Life and Health Sciences, Aston University and Department of Medical and Surgical Sciences, Magna Græcia University; 2 IMD Department, Queen Elizabeth Hospital, Birmingham, United Kingdom; 3 IMD Department, Birmingham Children’s Hospital, Birmingham, United Kingdom; 4 School of Life and Health Sciences, Aston University; 5 UK Newborn Screening Laboratories Network, Manchester, United Kingdom; 6 School of Life and Health Sciences, Aston University

**Keywords:** Adult PKU, metabolic diseases, cognitive impairments, executive functions and speed of processing, memory and learning

## Abstract

***Objective:*** Phenylketonuria (PKU) is an inherited metabolic disease which affects cognitive functions due to an inability to metabolize phenylalanine which leads to the accumulation of toxic by-products (Phe) in the brain. PKU can be effectively treated with a low phenylalanine diet, but some cognitive deficits remain. Studies have reported impairments, especially for processing speed and executive functions, but there is a lack of comprehensive assessment across cognitive domains. Moreover, it is important to establish outcomes in early treated adults with PKU (AwPKU) who have better metabolic control than groups previously reported in the literature. ***Method:*** We tested 37 AwPKU with an unprecedented number of tasks (*N* = 28) and measures (*N* = 44) and compared results with 30 controls matched for age and education. ***Results:*** We found (a) group impairments, particularly in tasks tapping speed of processing and complex executive functions; (b) high variability across participants, with a sizable number of AwPKU with completely normal performance (about 38%); (c) but also a sizable number of participants who were clearly impaired (about 24%); and (d) good performance in tasks tapping verbal learning, verbal memory and orthographic processing, indicating no generalized learning impairment. ***Conclusion:*** Our results indicate good outcomes, but also that deficits are still present with current treatment policies.

## General Scientific Summary

This study has advanced our understanding of the cognitive outcome in adults with phenylketonuria (AwPKU), highlighting the success, but also some limitations, of the clinical management. Our results indicate extreme variability across participants and across cognitive domains. As a group, AwPKU showed good performance in learning tasks, but reduced speed of processing and complex executive function deficits. This calls for a better understanding of what causes these impairments.

Phenylketonuria (PKU, ORPHA716) is a disorder caused by an inborn error in amino acid metabolism that, if untreated, causes severe mental retardation, microcephaly, seizures and behavioral problems (Blau, van Spronsen, & Levy, 2010). The metabolic defect underlying the classical form of PKU is a mutation in the gene coding for the enzyme phenylalanine hydroxylase (PAH), which is responsible for the conversion of the amino acid Phenylalanine (Phe) into *tyrosine*. The disrupted metabolism causes accumulation of Phe in the blood, toxic concentrations in the brain, and a reduced level of the amino acid tyrosine. The introduction of newborn screening programs and early treatment with a low-phenylalanine diet has prevented severe neurological damage and mental disability in patients with PKU, but number of studies have documented that even early treated patients suffer from a variety of mild cognitive impairments (see review later on). Still, there is a need of studies which document the performance of more recent cohorts of patients who have followed a stricter diet, and who are old enough to be assessed when brain development and cognitive development are completed. Linked to this need is the need of comprehensive assessments of cognitive functions across domains, because impairments may affect selective domains, and cognitive profile may differ at different developmental ages.

Most studies with early treated adult patients with PKU (from now on AwPKU) have reported impairments in speed of processing and executive functions, but only a very limited number of cognitive functions have been examined by each study (for a review see Christ, Huijbregts, de Sonneville, & White, 2010; Janzen & Nguyen, 2010; Moyle, Fox, Arthur, Bynevelt, & Burnett, 2007). Comparing outcomes across studies, however, is difficult. Functions are not always assessed in the same way and with the same tests and different cohorts of patients are bound to differ somehow for severity, age and socioeconomic status. This makes difficult to compare performance on the same functions across studies and to establish which functions, if any, are most affected by PKU. Moreover, although executive functions and visual attentional skills have been extensively studied, other functions have received less attention.

The lack of *comprehensive* neuropsychological assessments is one of the weaknesses in adult PKU research (but see Brumm, Azen, & Moats, et al., 2004 for an exception). Such assessments, instead, are crucial both for the clinical management of these patients and for a better understanding of the neurophysiological basis of the disease. To accurately evaluate the success of current treatment we need to identify how cognitive performance is affected *across* domains. Only in this way, can we provide patients with the correct educational and psychological support. In addition, we need to know if some functions are more affected than others to understand if the disease disrupts the functioning of specific brain areas and/or specific neurophysiological mechanisms (e.g., see the hypothesis of dopamine depletion following lack of precursor tyrosine vs. the hypothesis of a toxic effect on myelin; Anderson & Leuzzi, 2010; Anderson et al., 2007). Examining outcomes in adult patients is particularly important. Certain functions may only show a developmental delay, so that normal levels are reached even if at a later time. Alternatively, impairments may become more severe or even emerge only with time. Good baseline data is required to track possible disease progression with aging and to compare efficacy of possible treatments that are alternatives to dietary control (pharmacological treatments, enzyme replacement therapy: see Biomarin, 2016).

The seminal article by Brumm et al. (2004) provided some preliminary evidence of impairments across a variety of cognitive functions for a group of AwPKU. However, the number of patients was relatively limited (*N* = 22). More important, on average, the group had relatively poor metabolic control with an average blood Phe of 702 μmol/L before 10 years of age and >1000 μmol/L later on. These values are well above current European guidelines (above 18 years: 120–600 μmol/L; MacDonald personal communication, forthcoming at http://www.espku.org/who-we-are/european-guidelines) and American guidelines (American College of Medical Genetics and Genomics: 120–360 μmol/L throughout life; see Vockley et al., 2014). It is important, therefore, to establish if cognitive impairments are still present in AwPKU with better metabolic control reflecting more recent advice to maintain stricter blood Phe control.

In children with PKU, the deficits reported most often involve executive functions and a reduction in processing speed (Albrecht, Garbade, & Burgard, 2009; DeRoche & Welsh, 2008). As mentioned, studies of adult patients have reported similar deficits, but with some inconsistencies in their results, possibly, due to smaller number of participants in each study. We now briefly review these studies as well as studies which have assessed other cognitive functions, before outlining the plan of our study.

## Review of Literature

### Impairments in Executive Functions

Executive functions refer to a heterogeneous set of functions which are necessary to plan and complete tasks in spite of potentially distracting or irrelevant information (e.g., Alvarez & Emory, 2006; Diamond, 2013; Elliott, 2003; Miller & Cohen, 2001). We will review results according to ***type*** of executive function, subdivided into (a) complex functions including abstract reasoning and planning, (b) working memory, (c) sustained attention, and (d) inhibitory control. Although some researchers make fewer distinctions, we kept functions as separate as possible so that possible differences can be evaluated (see Diamond, 2013; Jurado & Rosselli, 2007; Latzman & Markon, 2010; Miyake et al., 2000, for similar subdivisions).

#### Complex executive functions

We have included in this category tasks requiring rule extraction, planning and flexibility/switching (e.g., Dubois, Slachevsky, Litvan, & Pillon, 2000; Stuss, & Benson, 1986). Typical tests used to assess these functions include the Wisconsin Card Sorting Test (WCST), which requires participants to infer, using feedback from the examiner, the right rule to sort cards into piles and, then, to change sorting criterion accordingly when feedback is changed and the Tower of Hanoi, a puzzle which requires forming a tower by moving circles from one peg to another, following rules. Impairments in the WCST have been reported by Brumm et al. (2004; *N* = 24; current Phe 157–1,713 μmol/L), by Nardecchia et al. (2015; *N* = 14 adults; current Phe not reported) and by Smith, Klim, Mallozzi, and Hanley (1996; *N* = 22; current Phe level 200–1,879 μmol/L), but not by Ris, Williams, Hunt, Berry, and Leslie (1994; *N* = 25; current average Phe 1,320 μmol/L). Channon, German, Cassina, and Lee (2004) also did not find any deficit (*N* = 20; current Phe 333–1,432 μmol/L), using a similar test (the Brixton test).

Other tasks that are commonly used to assess executive functions include the *Trail Making Test B and Verbal Fluency.* The Trail Making A requires connecting circles containing numbers, scattered randomly on a piece of paper, in ascending number order. The Trail Making B is more complicated because it requires alternating between numbers and letters. The difference between A and B is considered a measure of executive functions (Sánchez-Cubillo et al., 2009). AwPKU have shown normal performance in the *Trail Making B* (Moyle, Fox, Bynevelt, Arthur, & Burnett, 2007; *N* = 12, current Phe not reported, and Brumm et al., 2004).Verbal fluency requires participants to come up with as many words as possible in a unit of time consistent with a given criterion (beginning with a given letter or belonging to a given semantic category). Verbal fluency is believed to be part of executive function because it requires *planning* a systematic search of the lexicon to avoid wasting time on exhausted areas (Alvarez & Emory, 2006; Baldo, Shimamura, Delis, Kramer, & Kaplan, 2001; Latzman & Markon, 2010). Verbal fluency was reported to be impaired in AwPKU by Channon et al. (2004) and Brumm et al. (2004), but not by Moyle, Fox, Bynevelt, et al. (2007).

#### Working memory/short-term memory

This is the capacity which allows us to keep in mind, manipulate and refresh the information necessary to complete a task (see Baddeley, 1986; McCabe, Roediger, McDaniel, Balota, & Hambrick, 2010). Typical tasks used to tap this function are forward and backward digit span tasks (where sequences of digits have to be repeated back in the order in which they were presented or in reverse order). The Corsi Block test is a typical visuospatial working memory task (it requires participants to touch a sequence of blocks in the same order in which they were touched by the examiner or in reverse order). Most studies have reported working memory to be impaired in AwPKU (Bik-Multanowski, Pietrzyk, & Mozrzymas, 2011; Brumm et al., 2004; Channon et al., 2004; Channon, Goodman, Zlotowitz, Mockler, & Lee, 2007; Channon, Mockler, & Lee, 2005; but see Moyle, Fox, Bynevelt, et al., 2007 for negative results).

#### Sustained attention

This is the capacity to keep in mind target information for a sustained amount of time in spite of interfering information being presented (Sarter, Givens, & Bruno, 2001; Wilkins, Shallice, & McCarthy, 1987). Typical tasks used to tap this function are the continuous performance task or the rapid visual information processing task, in which participants are presented with sequences of digits and must detect target sequences. Sustained attention has been reported to be impaired in AwPKU (Bik-Multanowski et al., 2011; Schmidt at al., 1994; Weglage et al., 2013).

#### Inhibitory control

This refers to the ability to flexibly modify answers and inhibit inappropriate responses depending on task demands (e.g., Latzman & Markon, 2010; Miyake et al., 2000). A prototypical task tapping this ability is the *Stroop Test* where participants have to name the ink color of words whose meaning refers to a different color (say “yellow” for the word “red” written with yellow ink), thus, suppressing the tendency to read the word. Impairments have been reported in children with PKU, but only sporadically in adults (for a review of children studies, see DeRoche & Welsh, 2008; AwPKU impaired in Sundermann et al., 2011, *N* = 17; current Phe average 1140 μmol/L; not impaired in Brumm et al., 2004; Feldmann, Denecke, Grenzebach, & Weglage, 2005). No impairment has also been reported in other tasks tapping inhibitory control such as the *Hayling Sentence Completion Test* which involves completing sentences as quickly as possible with nonsensical words (Channon et al., 2004) and the *Flanker Test* in which participants have to respond to the direction of a central arrow, ignoring flanker arrows that may point in opposite ways (Channon et al., 2005).

### Impairments in Speed of Processing

As evident from the review above, results in the assessment of executive functions have been mixed, in spite of groups being similar for Phe levels at the time of testing (although historical Phe are not always reported and could be responsible for variations). Negative findings are particularly evident for inhibitory control, which is considered a typical executive function. These mixed results have lead some authors to suggest that the main residual deficit in AwPKU in one of speed of processing with deficits in executive tasks being largely resolved (e.g., Channon et al., 2005, 2007; Moyle, Fox, Bynevelt, et al., 2007; Feldmann et al., 2005). Channon et al. (2005, 2007), for example, compared the performance of 25 AwPKU (concurrent Phe level 221–1233 μmol/L) and 25 matched controls on two executive tasks tapping working memory (*n*-back; Braver et al., 1997) and inhibition (Flanker inhibitory task), and two nonexecutive tasks (one involving learning an object location, and one involving categorizing objects for shape or function). AwPKU showed only a speed deficit in the working memory task and performed normally in the other tasks, prompting the conclusion that the only remaining deficit is one of speed. This conclusion, however, may be premature. Although inhibitory control may normalize in adulthood, other executive functions may remain suboptimal.

### Impairments in Other Cognitive Functions

#### Visuomotor coordination

Impairments have been reported both in children and adults, but it is unclear whether deficits are primary or a consequence of a more general speed reduction, because performance is generally tested with timed tasks. In adults, deficits have been found using tasks like the *Grooved Pegboard Test*, which requests participants to insert pegs into small holes with one hand, as quickly as possible (see Griffiths, Paterson, & Harvie, 1995; Pietz et al., 1998, but see Brumm et al., 2004, for negative results), but not with the *Digit-Symbol Coding Test* of the WAIS (Brumm et al., 2004) where participants have to write numbers below symbols according to given letter-symbol pairings. Arguably, digit-symbol coding requires a variety of skills (keeping in mind the number-symbol pairings and switching back on forth between the pairings and the symbols under which the numbers have to be written; see also Davis & Pierson, 2012). However, it has a clear visuomotor component because visual information has to guide the motor response and, for this reason, has been considered by Brumm et al. (2004) to tap mainly visuomotor coordination.

#### Language processing, and memory and learning

Impairments in these domains have been reported less frequently in early treated PKU children (for a review see Janzen & Nguyen, 2010). In AwPKU, results are inconsistent. Typical tasks include learning a list of words across trials (e.g., Rey Word Test), learning the positions of shapes (e.g., paired associated visual learning) or drawing a complex picture from memory (Rey–Osterrieth Complex Figure). No memory impairment was reported by Griffiths et al. (1995) in the verbal domain and by Channon et al. (2004) in the visuospatial domain. In contrast, memory impairments have been reported by Brumm et al. (2004) and Smith et al. (1996) across domains and by Griffiths et al. (1995) in the visuospatial domain.^1^ In addition, Brumm et al. (2004) have reported impairments in expressive naming (Boston Naming Test), but not in receptive vocabulary (Peabody Picture Vocabulary Test).

## Plan of Study and Data Analyses

In sum, for most cognitive functions including complex executive functions, visuomotor coordination, language processing, and memory and learning, results have been mixed. Deficits have been reported more consistently for working memory and sustained attention and normal performance for inhibitory control. Some authors have suggested that only deficits of speed of processing characterize AwPKU.

Our study will establish the cognitive profile of a group of 37 AwPKU with better metabolic control than reported in previous studies (average 432 μmol/L, in childhood; <850 μmol/L later on). Our study is similar to Brumm et al. (2004) in assessing a large number of functions and using a variety of tests for each function to reduce error. Differently from Brumm et al., we have decomposed executive functions in a number of subtypes, assessed visuospatial attention as a separate domain, and included assessment of orthographic skills.

Besides performance in individual tasks and functions, we will consider two general measures of performance across domains: (a) an overall standard score, which averages across cognitive tests and measures (RTs and accuracy); and (b) the proportion of impaired scores. This second measure is also very important because good performance in a number of tasks may counterbalance and mask severe impairments in others and result in an overall score which is normal or close to normal. Therefore, we will also consider, for each PKU participant, the number of cognitive domains/measure where severe difficulties are encountered. Even control participants are anticipated to demonstrate some poor scores, but this number may be much higher in AwPKU. For these measures (overall standardized score and rate of impaired scores) we will report both group averages and proportions of individuals impaired. To be conservative, we will consider impaired scores which deviate 2 or more standard deviations from the mean of a control group that we tested for comparison and was matched to the AwPKU for age and education (z scores = >2). We will consider scores which are within 0.5 standard deviations from the control mean (z scores = <.05) to be clearly normal.

## Method

### Participants and Procedure

Thirty-seven early treated patients with classical PKU were recruited from a pool of patients currently followed by the Department of Inherited Metabolic Disorders at the Queen Elisabeth Hospital in Birmingham who had been continuously treated with a low-phenylalanine diet since birth. Diagnosis was through newborn screening conducted at 5–7 days after birth. At the time of testing 7 patients were on an unrestricted diet and 30 on a low phenylalanine diet. We invited all early treated patients attending the clinic to participate, plus patients who were still contactable, but no longer in regular follow-up. All individuals who responded to the invitation were tested. No patient had been diagnosed with any psychiatric disorder. Data on historical Phe levels were obtained from the PKU database at The Clinical Chemistry Department at Birmingham Children’s Hospital.

The PKU participants were compared with a group of 30 healthy control participants matched for age, gender, and educational status. They were recruited through the Aston University volunteering website.

Participants were tested in a quiet room in two separate testing sessions, each lasting approximately 3 hours. Blood Phe concentrations were measured prior to each testing session to determine current Phe levels. The research was approved by the NHS and Aston University Ethics committees. All participants gave voluntary informed consent to take part. All efforts were made to administer all tasks to all participants, but some data points are missing because not everybody returned for the second session (see Tables 1–3 for exact numbers). Whenever possible we report performance in terms of error rates so that like for speed measures higher scores indicate lower performance.[Table-anchor tbl1][Table-anchor tbl2][Table-anchor tbl3]

### Tasks

IQ was measured using the Wechsler Abbreviated Scale of Intelligence (WASI; Wechsler, 1999), which includes the following subtests: Vocabulary, Block Design, Similarities, and Matrix Reasoning. These subtests are similar in format to their WISC–III and WAIS–III counterparts. They are those with the highest loadings on general intellectual functioning (Wechsler, 1991, 1997) and allow estimates of both Verbal and Performance IQ. In addition, participants were given an extensive neuropsychological battery. Tasks are briefly described below. A more extensive description is provided in the supplementary materials.

#### Visuospatial attention

This included measures from 6 tasks: (a) *Simple Detection*: Press a response button as soon as a ladybird appeared on the screen; (b) *Detection with Distractors*: Press a button whenever a ladybird appeared on the screen alone or with a green bug; in the second part of the task the instruction was changed to press a button whenever a green bug appeared on the screen alone or with a ladybird; (c) *Choice Reaction Time*: Press either a left or right response key consistent with the direction of an arrow centrally presented; (d) *Feature Search*: Detect a target among distractors not sharing features by pressing a ‘yes’ or ‘no’ button (e.g., a red ladybird among green bugs); and (e) *Conjoined Search*: Detect a target among distractors sharing features (e.g., red ladybird among red bugs and green ladybirds). Both RTs (reaction time (RT) from now on) and accuracy measures (error rates) were taken.

#### Visuomotor coordination

This included measures from 2 tasks: (a) *Grooved Pegboard Test*: Put pegs into the holes of a board using only one hand as quickly as possible and (b) *Digit Symbol Task*: Fill as many boxes as possible with symbols corresponding with numbers in 90 s.

#### Complex executive functions

This included measures from 4 tasks which, to be completed involve planning, flexibility and abstract thinking. First, *The Wisconsin Card Sorting Test*, (64 Cards Version) in which participants have to discover the rules to match cards from a deck with four reference cards according to shape, number and color using feedback. Flexibility is required when the sorting rule is changed unknown to the participant and the new rule has to be discovered. A number of detailed measures are reported in Table 3 below. To compute an aggregate score for ‘Complex executive functions’ we used total number of errors. Second was difference in speed between *Trail Making Test B-A* (A involves connecting circles containing numbers in ascending order as quickly as possible; B also involves connecting circles in ascending order but alternating between number and letters). Only speed measures are taken because errors are rare. Third, for *Fluency*. For letter fluency, participants generate as many words as possible starting with a given letter in one minute of time (letters: C, F and L). For semantic fluency, they generate name of animals. We only considered semantic fluency in the aggregate score for ‘Complex Executive functions’ as better reflecting the ability to carry-out an efficient lexical search. And finally, for *The Tower of Hanoi* puzzle, participants move a number of rings of different sizes across three pegs to form a tower on the last peg following specific constraints. Our score is based on the percentage of solved trials of different complexity (3, 4, 5 rings).

#### Inhibitory control

This included measures from 2 tasks: (a) *Stroop* interference: Difference in time and errors between reporting the ink color of words where the color of the ink was incongruent with the meaning of the word; “red” written with yellow ink, or congruent, “red” written with red ink; (b) *Semantic Interference*: Differences in naming between the first and last exemplar of a series of semantically related nouns in terms of RT and errors.

#### *Short-term Memory/Working Memory*

This included measures from 3 tasks: (a) *Digit Span*: Repeat a sequence of digits spoken by the examiner, soon after presentation; (b) *Nonword Repetition*: Repeat a sequence of nonwords spoken by the examiner, soon after presentation; and (c) the *Corsi Block Tapping Test*: The examiner taps a sequence of blocks and the participant has to reproduce the sequence in the same order.

#### *Sustained Attention*

Percentage correct from the Rapid Visual Information Processing task: detect three target sequences of 3 digits by pressing the response key when the last number of the sequence appears on the screen.

#### Orthographic Language

This involved measures from 6 tasks: (a) and (b) *Word and Nonword Reading:* Read as fast as possible an English or a made up word and both RT and accuracy measures (error rates) are taken; (c) and (d) *Word and Nonword Spelling*: Spell words/nonwords to dictation; (e) *Phoneme Deletion*: Delete a sound from a word (e.g., powder; /d/ > power); and (f) *Spoonerisms*: Exchange the initial sounds of two words to produce two different words (e.g., bad-sin > sad-bin). These last two tasks were included because performance shows strong correlations with orthographic skills (Landerl, Wimmer, & Frith, 1997; Romani, Tsouknida, & Olson, 2015).

#### Spoken Language

This included measures from four tasks: (a) *Picture Naming*: Name a picture as fast as possible; (b) *Color naming*: Name as fast as possible the ink color of three *X*s or colored words; only the congruent condition is considered here, where the color of the ink matched the meaning of the word, for example, “red” written with red ink. Both RT and accuracy measures (error rates) were taken; (c) *Similarities* from the WASI: Describe how similar in meaning two words are; and (d) *Vocabulary* from the WASI: Define a word.

#### Verbal Memory and Learning

This included measures from 2 tasks: *The Rey Auditory Verbal Learning Test* (learning, immediate recall and delayed recall of a list of 15 words) and *Paired Associates Verbal Learning* (learning the association between a made-up word and the picture of an object or animal).

#### Visual Memory and Learning

This included measures from 2 tasks: (a) *Delayed Matching to Sample*: Recognize a previously seen pattern among distractors and (b) *Paired Associates Visual Learning*: Learn to associate shapes with locations.

## Results

Demographic and Phe data, as well as general cognitive performance, are reported in Table 1. Metabolic values are reported in three age bands: childhood (0–10 years old), adolescence (11–16 years old), and adulthood (17 years to present), as well as at testing time and across the lifetime. We report metabolic control both in terms of mean Phe level and Phe fluctuation. The Phe level in each band was calculated by averaging the Phe medians for each year in the band; the Phe fluctuation was calculated by averaging standard deviations from median values (*SD*) for each year in the band. For each AwPKU, the current Phe was calculated averaging Phe level at Session 1 and Phe level at Session 2. Across the group, Phe levels were better controlled in childhood; diet was progressively relaxed after early childhood with increasing blood Phe levels.

### General Cognitive Performance and Variability Between Participants

Overall, AwPKU had a full scale IQ in the average range (only one impaired patient), but significantly lower than matched controls (see also DeRoche & Welsh, 2008; and Moyle, Fox, Arthur, et al., 2007, for a meta-analysis). Considering the whole neuropsychological assessment together (44 measures), the mean AwPKU *z* score was only slightly below the control average (*z* score = **0.5**). However, a different picture emerged when performance with individual tasks was considered. Specifically, for each participant we calculated the percentage of impaired measures (a measure was considered impaired if it was >2 *z* score from the mean of our control group) on the overall number of available measures. On average, 4% (± 3.7%) of measures were impaired in controls versus 13% (± 15.1%) AwPKU. In 17/37 (46%) of PKU participants, the rate of impaired measures was significantly above control average. Moreover, 9 AwPKU (**24%** of the PKU sample**)** showed *both* a pathological rate of impaired measures (>2 *z* scores) *and* an overall *z* score below average (= >1 *z* score), thus showing overall a clear impairment. In contrast, **38%** of PKU participants (14/37) showed a completely normal profile with *z* scores = < 0.5, both for rate of impaired measures and overall average performance.

AwPKU with impaired (*N* = 9) versus normal (*N* = 14) performance did not have significantly different Phe levels during childhood (μmol/L 469 vs. 485; *t*(1,18) = −1.21; *p* = .90), but differed significantly for metabolic control in adolescence (μmol/L 659 vs. 944; *t*(1,19) = −2.2.; *p* = .04) and adulthood (μmol/L 782 vs. 1128; *t*(1,21) = −3.3; *p* = .003) and for current Phe (μmol/L 713 vs. 1,059; *t*(1,20) = −2.7; *p* = .01). They also differed in terms of Phe variability (in terms of average *SD* per year) in adolescence (μmol/L *SD* 148 vs. 214; *t*(1,19) = −3.5; *p* = .002) and through the life span(μmol/L *SD* 162 vs. 212; *t*(1,21) = −2.5; *p* = .02), but variability was not different at other times.

**Figure 1 fig1:**
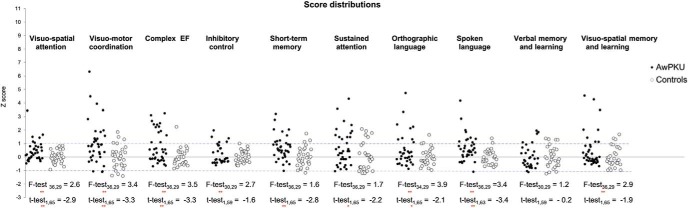
Distribution of scores for the Control and PKU groups across speed tasks. Each point is the performance of an individual participant; *t* tests assess differences in average performance between groups; Variance ratio *F* test assess differences in variability. Tasks were aggregated across domains by averaging *z* scores across tasks/measures as follows: (a) Visuospatial attention: RTs in *Simple Detection,* and RT and accuracy in *Detection with Distractors, Choice Reaction Time, Feature Search, Conjoined Search*; (b) visuomotor coordination: *Grooved Pegboard Test, Digit symbol*; (c) complex executive functions: *WCST score, Tower of Hanoi scores, Semantic fluency, Trial Making B-A*; (d) inhibitory controls: *Stroop interference effect; Semantic interference effects* (both RTs and accuracy); (e) STM (short-term memory): *Digit Span, Nonword repetition, Corsi Span*; (f) Sustained attention: *Rapid Visual Information Processing -RVP*; (g) Orthographic Language: RT and accuracy in *words and nonword reading,* accuracy in *word and nonword spelling, spoonerisms, and phoneme deletions*; (h) Spoken Language: RT and accuracy in *picture naming* and *color naming*, *Vocabulary WASI, Similarities WASI*; (i) verbal memory and learning: *The Rey Auditory Verbal Learning Test* and *Paired Associates Verbal Learning*; (j) Visuospatial memory and learning: *Delayed Matching to Sample* and *Paired Associates Visual Learning*. * *p* < 0.05. ** *p* < 0.01. Please see the online article for the color version of this figure.

Figure 1 shows that although, in each domain, most scores of AwPKU fall within 1 *SD* of the control mean, there are a substantial number of AwPKU performing below average. The only domain where this does not occur is verbal memory and learning where the distribution of scores appears similar in the PKU and control group.[Fig-anchor fig1]

### Variability Across Domains

Performance of the PKU and control groups on individual tasks is shown in Tables 2–4. Table 2 shows results for visuospatial attention, visuomotor coordination and visuospatial memory. AwPKU are systematically impaired in tasks requiring visuospatial attention except for the easiest tasks (Simple Detection and Detection with Distractors). No significant impairments are seen in accuracy measures, but this could be due to lack of sensitivity of these measures (performance being close to 100% correct across groups). Performance on the tasks tapping visuospatial memory and learning failed to reach significance when the tasks were considered individually, but was marginally lower when scores are aggregated, as shown in Figure 1. Performance on tasks tapping visuomotor coordination is impaired, consistent with previous results (see Griffiths et al., 1995; Pietz et al., 1998).[Table-anchor tbl4]

Table 3 shows results for executive functions. There is a contrast between tasks requiring higher order executive functions where differences are highly significant (WCST, Tower of Hanoi, Semantic Fluency) and tasks which arguably require less planning and monitoring, where no significant difference is present (the Corsi Block, the Letter Fluency and the Trial making B-A). There is also no impairment in tasks tapping inhibitory control, consistent with previous studies (Brumm et al., 2004; Channon et al., 2004, 2005). Finally, there is a significant impairment of sustained attention (see also Bik-Multanowski et al., 2011; Schmidt at al., 1994; Weglage et al., 2013). It is important to note that impairments are seen in executive functions even if tasks are untimed or have a minimal timing component. The WCST is untimed. The Tower of Hanoi is timed (maximum of 6 min for each trial), but participants rarely fail due to time constraints. Verbal fluency has a speed element because scores are based on the number of words produced in a fixed unit of time. However, the quality of the lexicon (its size and structure) and the strategies adopted to search it are more important than speed of access per se. Similarly, the task tapping sustained attention involved detection of number sequences within time constraints. However, the detection window included two digits following the last digit of the target sequence (totaling 1,800 ms) which should minimize difficulties with speed. The fact that, as a group, AwPKU show impairments in all of these tasks argues against the hypothesis that, in adulthood, deficits *only* involve a reduction of speed of processing.

Table 4 shows results for language tasks. Performance on tasks probing orthographic knowledge is good. Speed in word and nonword reading is impaired, consistent with a generalized reduction in speed of processing, but accuracy is good, as is performance in spelling, spoonerisms and the phoneme deletion task. Word spelling is numerically better than in the controls. Note also that word reading speed was relatively less impaired than nonword reading speed, group by task interaction: F_(1, 59)_ = 10.5, *p* < .01; partial eta-squared = .2, consistent with the deficit being more in components involved in perceptual analysis than in lexical access. Accuracy in picture naming was good. More taxing tasks probing vocabulary (vocabulary and similarity subtests of the WASI) were impaired, but these tasks have a strong reasoning component which could be responsible for the deficit. Finally, performance was excellent in memory and learning tasks. Combined, these results indicate excellent ability to learn in our AwPKU group.

**Figure 2 fig2:**
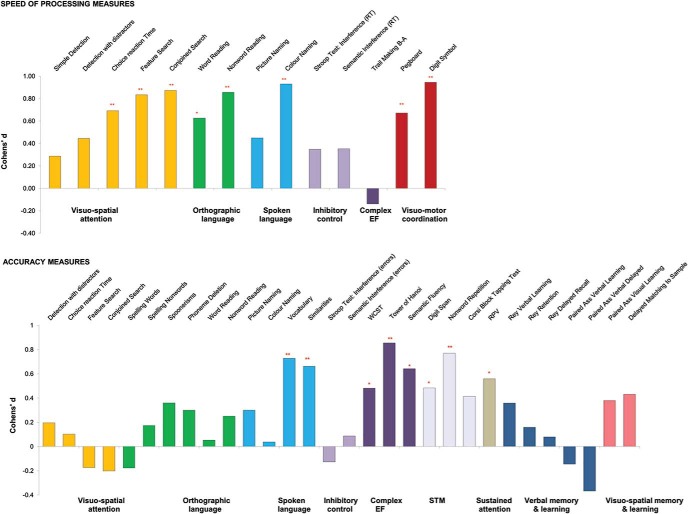
Differences in performance of the PKU group from the control group in terms of Cohen’s *d* across functions; Cohen’s *d* = difference between the two groups averages divided by the pooled *SD*. Asterisks indicate a significant difference between AwPKU and Control. * *p* < 0.05. ** *p* < 0.01. See the online article for the color version of this figure.

To better compare the relative severity of impairments with speed and accuracy measures Figure 2 reports severity of PKU impairment in terms of Cohen’s *d* for different domains. The Cohen’s *d* is the difference between the mean performance of PKU and control groups divided by the pooled standard deviation (see Rosenthal, 1994). In our case, the higher the score the bigger the difference between the two groups.[Fig-anchor fig2]

It is clear that speed of processing is systematically impaired across domains with significant differences in most tasks and, on average, a medium Cohen’s *d* (.**58**). Accuracy measures return a lower average Cohen’s *d* (**.26**), but there is high variability across domains, making this figure poorly representative. The Cohen’s *d* remains high for tasks which are taxing and require complex skills, including *planning* and *switching* (WCST, Tower of Hanoi, and Fluency), *monitoring* (short-term memory [STM[, Sustained attention) and *verbal reasoning* (Similarities, Vocabulary of WASI).

## General Discussion

We have carried out a comprehensive neuropsychological investigation in a group of 37 AwPKU with relatively good metabolic control since childhood and 30 matched controls. This has advanced our understanding of the effect of PKU on cognition by identifying which cognitive functions are generally spared and which continue to be affected and by providing a more complete picture and the severity and variability of impairments in this population.

### Cognitive Impairments

We have shown significant and widespread cognitive impairments, even for a group of AwPKU who have had better metabolic control than reported in previous studies. Europe recommendation for target Phe levels have been very variables across countries (Blau et al., 2010). New forthcoming European guidelines from ESPKU suggest a target of <360 μmol/L in childhood and <600 μmol/L afterward. Our group does not completely meet these guidelines but comes closer than previously reported groups (e.g., concurrent Phe level in our group = 720 μmol/L compared with most groups were it is ∼1,000 μmol/L; see Introduction). Testing groups with better control will continue to be important in the future to provide firm evidence of the success and limitations of current treatment.

In our PKU group, impairments were present across a variety of domains showing that, although reduction in speed of processing is a factor underlying cognitive difficulties, impairments are also present in tasks with no or a minimal speed component. Impairments were present especially in complex tasks involving reasoning, planning and monitoring, and, more generally, in tasks which required the orchestration of a variety of skills. In contrast, our PKU group did not show learning difficulties and performance in tasks of verbal and visual learning and memory was very good. Performance in spelling, another task which relies heavily on learning and on the capacity to store information, was also excellent, as was performance on tasks which relied heavily on orthographic knowledge (phoneme deletion, spoonerisms). Good performance here is not due to lack of test sensitivity. On the contrary, these learning and orthographic tasks are challenging even for control participants and among the most sensitive tasks to detect impairments in other clinical populations such as adults with developmental dyslexia (e.g., see Romani, Di Betta, Tsouknida, & Olson, 2008). The good performance on these tasks may also reflect the good support received by our participants with PKU and/or acquired coping strategies. AwPKU may have learned to rely more on stored knowledge to compensate for their weaknesses in speed of processing.

#### Speed of processing

In PKU studies, speed of processing has, generally, been measured with visuospatial tasks where participants are asked to detect targets in different types of displays. In our study, we have also shown impairments in tasks involving picture naming, and word and nonword reading. Instead, we have shown no impairment in tasks which require speed but minimize cognitive processing by only requiring a response when a given stimulus appears on the screen (Simple Detection and Detection with Distractors). This result is important because it shows that AwPKU experience *cognitive* slowing, but not a *peripheral* reduction in motor speed. If PKU caused a generalized speed deficit, impacting equally on the central and the peripheral nervous system, then a task requiring simple target detection (particularly in the absence of distractors) should be especially affected. Here, the main factor affecting performance should be the speed with which action potentials travel along the long and heavily myelinated axons of the motor neurons. If myelination of these axons were degraded, speed would be impaired. Our results, instead, indicate that slowing occurs at the cognitive level, consistent with the toxic effects of high Phe levels on the oligodendroglia in the central nervous system (see Möller et al., 2003; Moyle, Fox, Arthur, et al., 2007), but not on the Schwann cells in the peripheral nervous system (see Joseph & Dyer, 2003). Consistent with our results, a recent meta-analysis (Albrecht et al., 2009) reported choice RT to be the speed test most sensitive to phenylalanine concentrations.

#### Impairment of executive functions

We have found significant impairments in tasks tapping complex executive functions, in particular functions involving planning/cognitive flexibility (Tower of Hanoi, WCST), monitoring (STM, sustained attention) and verbal reasoning (vocabulary and similarities from the WASI). Other tasks which are impaired, like the digit symbol task, also require a combination of complex skills including switching and monitoring (for similar impairments in higher order executive functions see Brumm et al., 2004; Smith et al., 1996; Nardecchia et al., 2015). Instead, we have found no impairments in tasks taping inhibitory control consistent with previous results (Brumm et al., 2004; Channon et al., 2004, 2005). This pattern is the opposite of what is seen in children and adolescents where inhibitory control seems to be particularly affected, whereas working memory and planning are less affected (see DeRoche & Welsh, 2008). This may be explained by the different developmental trajectory of these functions. Inhibitory control develops in childhood and peaks in early adolescence whereas planning and monitoring functions may continue to develop through adulthood (see Anderson, 2002; Best & Miller, 2010; Romine & Reynolds, 2005). It is possible that some functions, like inhibitory control, only show a developmental delay, but eventually catch up. Instead, other executive functions involving different skills (monitoring) or more complex skills (planning, reasoning) either plateau later on or, more likely, will continue to show deficits because they are more sensitive to impairment.

### Variability

We have shown *extreme* variability across participants. Only 2/37 = 5.4% of our PKU participants showed a *severe* impairment in terms of average performance (with overall performance being below 2 *SD* from the mean), but a much higher number showed an abnormal cognitive profile in terms of proportion of impaired measures (46%). Overall about 1/4 of our sample (24%) displayed a clear cognitive impairment (in terms of both average performance and cognitive profile), whereas 38% performed as well as the controls with *z* scores always = <.05. It is important to note that AwPKU with a normal versus impaired cognition significantly differed for metabolic control (Phe average in adolescence and adulthood and Phe variability across the lifespan), underscoring the importance of maintaining control at all ages.

## Conclusion

We have carried out a cognitive assessment of a sample of adults with PKU using an unprecedented number of tasks across domains. Our results show the importance of carrying out such a comprehensive assessment. This has allowed us to gather important information about the type, severity and distribution of cognitive impairments in AwPKU. Regarding type of impairments, we have found that even in a group of relatively well controlled AwPKU there are still impairments in complex executive functions and responses are slower across domains. Learning abilities, however, were well preserved and a reduction in speed was limited to cognitive tasks with no effects on peripheral motor speed. This indicates that not all cognitive functions are equally susceptible to the toxic effect of Phe. In terms of severity and distribution of impairments, our results show extreme variability with about 1/4 of AwPKU obtaining a completely normal cognitive profile, but about half showing significant impairments in at least some functions. These results highlight the success, but also the limitations of the clinical management received by these patients. A large number of AwPKU reach performance which is indistinguishable from their peers. This should be reassuring for them, their families and the professionals involved in their care. However, there are still impairments in a large number of AwPKU which may reduce their career potential and economic prospects. This variability calls for a better understanding of what causes these impairments and for better treatment. A possible cause of variability is, of course, dietary adherence and current and historical levels of Phe. We have seen that AwPKU with normal versus impaired cognitive performance differ significantly in level of metabolic control. Although our AwPKU showed on average a better metabolic control than that reported in previous studies, forthcoming European guidelines or current American guidelines are even more stringent and could result in still better outcomes. Further studies should investigate how metabolic control at different ages may impact cognition and explore possible differences across cognitive domains (see Romani, Palermo, MacDonald, Limback, Hall, & Geberhiwot, 2017).

## Supplementary Material

10.1037/neu0000337.supp

## Figures and Tables

**Table 1 tbl1:** Demographic Information, Metabolic Control, and General Cognitive Performance for 30 Control and 37 PKU Participants

Variable	Controls		PKU participants		Controls vs. PKU
	*M*	*SD*	*M*	*SD*	
Age	**27.6**	7.4	**27.5**	7.3	*t*_(1, 65)_ = 0.08; *p* = .94
Education years	**15.2**	1.7	**14.4**	1.9	*t*_(1, 65)_ = 1.6; *p* = .12
Gender (M/F)	**10/20**		**13/24**		χ_(1)_^2^ = .02; *p* = .88
Childhood (mean *N* obs. = 197; *SD* = 165)					
Phe average			**432**	243	
Phe fluctuation (*SD*)			**205**	63	
Adolescence (mean *N* obs. = 77; *SD* = 70)					
Phe average			**721**	340	
Phe fluctuation (*SD*)			**157**	58	
Adulthood (mean *N* obs. = 65; *SD* = 74)					
Phe average			**802**	324	
Phe fluctuation (*SD*)			**137**	68	
Lifetime (mean *N* obs. = 340; *SD* = 241)					
Phe average			**634**	291	
Phe fluctuation (*SD*)			**164**	58	
Current Phe			**720**	343	
Cognitive performance					
FIQ	**113.8**	10.9	**103.9**	14.3	t_(1, 65)_ = 3.1; *p* = .003
VIQ	**112.2**	10.2	**102.3**	12.9	t_(1, 65)_ = 3.1; *p* = .001
PIQ	**112**	11.7	**104.5**	15.1	t_(1, 65)_ = 2.2; *p* = .03
Overall *z* score	**.0**	.3	**.5**	.7	t_(1, 65)_ = −3.8; *p* < .001
Impaired participants					
*N*	**0**		2		
%	**0**		5.4		
Impaired measures (%)	**3.8**	3.7	**13.2**	15.1	*t*_(1, 65)_ = −3.4; *p* = .001
Impaired participants					
*N*	**1**		**14**		
%	**3.3**		**37.8**		χ_(1)_^2^ = 11.35; *p* < .001
*Note.* Blood Phe measured in μmol/L. Impaired= > 2 *z* scores from control means. PKU = phenylketonuria; Phe = Phenylalanine.					

**Table 2 tbl2:** Speed and Accuracy in Visuospatial Attention, Visuomotor, and Visuospatial Memory Tasks in Control and PKU Participants

Domain/task	Controls		PKU participants					Controls vs. PKU
	*M*	*SD*	*M*	*SD*	*N*	% *z* = > 2	% *z* = > 1	*t* and *p*
Visuospatial attention								
Simple detection (RT – ms)	**316**	56.9	332	52.9	31	6.5	16.5	*t*_(1, 59)_ = −1.1; *p* = .27
Detention with distractors								
RT − ms	**407**	72.2	**438**	70.6	31	3.2	22.6	*t*_(1, 59)_ = −1.7; *p* = .09
% errors	**.6**	.6	**.8**	1.1	31	6.5	19.4	*t*_(1, 59)_ = −.8; *p* = .45
Choice RT								
RT − ms^a^	**281**	31.3	**307**	42	37	16.2	32.4	***t*_(1, 65)_ = −2.8; *p* < .01**
% errors	**.4**	.8	**.5**	.8	37	8.1	8.1	*t*_(1, 65)_ = −.4; *p* = .68
Feature search								
RT − ms^a^	**498**	71.7	**606**	167.6	31	32.3	41.9	***t*_(1, 59)_ = −3.2; *p* < .01**
% errors	**2**	2.3	**1.6**	2.6	31	6.5	16.1	*t*_(1, 59)_ = .7; *p* = .50
Conjoined search								
RT − ms^a^	**841**	126.3	**1,008**	239.7	31	25.8	38.7	***t*_(1, 59)_ = −3.4; *p* < .01**
% errors	**3.1**	4.4	**2.4**	2.7	31	0	3.2	*t*_(1, 59)_ = .8; *p* = .43
Visuomotor coordination								
Pegboard (time – seconds)^a^	**59.2**	5.1	**65.1**	11.7	37	18.9	43.2	***t*_(1, 65)_ = −2.6; *p* = .01**
Digit symbol (% errors in 90 s)^a^	**27.1**	10.2	**37.3**	11.3	31	19.4	41.9	***t*_(1, 59)_ = −3.7; *p* < .01**
Visuospatial memory and learning								
Paired Associate Verbal Learning (% errors)	**1.9**	1.9	**3.1**	3.82	31	12.9	16.1	*t*_(1, 59)_ = −1.5; *p* = .15
Delayed matching to sample (% errors)	**10.5**	8.3	**14.1**	8.5	37	10.8	29.7	*t*_(1, 65)_ = −1.7; *p* = .09
*Note*. Percentage of Phenylketonuria (PKU) participants with *z* score = >2 (expected % 2.3) is also reported. RT = reaction time.								
^a^ Tasks where there is a significant difference with controls.								

**Table 3 tbl3:** Executive Function Tasks in Control and PKU Participants

Domain/task	Controls		PKU participants					Controls vs. PKU
	*M*	*SD*	*M*	*SD*	*N*	% *z* = > 2	% *z* => 1	*t* and *p*
Complex executive functions								
WCST								
Total errors^a^	**10.8**	4.9	**14**	8.2	37	24.3	27	***t*_(1, 65)_ = −2.0; *p* = .05**
Perseverative responses	**6.8**	4.4	**8.1**	5.1	37	10.8	27	*t*_(1, 65)_ = −1.1; *p* = .27
Perseverative errors	**6.1**	3.3	**7.4**	4.3	37	10.8	24.3	*t*_(1, 65)_ = −1.4; *p* = .16
*N* of completed categories^a^	**4.4**	.9	**3.9**	1.2	37	13.5	32.4	***t*_(1, 65)_ = 2.1; *p* = .04**
Trail making: B–A (Time – seconds)	**20.7**	13.3	**19**	10.8	37	5.4	10.8	*t*_(1,65)_ = −.6; *p* = .57
Tower of Hanoi (% of not solved trials)^a^	**2.9**	7.9	**20.6**	28.2	27	33.3	48.1	***t*_(1, 52)_ = −3.1; *p* < .01**
Verbal fluency								
Letter (correct answers)	**41.8**	12.9	**35.9**	11.4	37	2.7	27	*t*_(1, 65)_ = 1.9; *p* = .06
Semantic (correct answers)^a^	**25**	5	**21.4**	6.2	37	10.8	37.8	***t*_(1, 65)_ = 2.6; *p* = .01**
Inhibitory control								
Stroop Test: Interference								
RT – ms	**94.3**	51.9	**111.3**	86.7	30	16.7	23.3	*t*_(1, 58)_ = −1.4; *p* = .18
% errors	**.8**	1	**.5**	1.1	30	3.3	13.3	*t*_(1, 58)_ = .5; *p* = .63
Naming: Semantic interference								
Position 5–1 (ms.)	**145**	62.9	**173**	94.6	30	16.7	33.3	*t*_(1, 58)_ = −1.4; *p* = .18
Position 5–1 (% errors)	**1.1**	1.6	**1.3**	2	31	9.7	16.1	*t*_(1, 59)_ = .3; *p* = .74
Short-term memory								
Digit span^a^	**6.5**	.9	**6.1**	1	37	10.8	35.1	***t*_(1, 65)_ = 2.0; *p* = .05**
Nonword repetition (% errors)^a^	**39.3**	10.4	**48.2**	12.5	31	19.4	45.2	***t*_(1, 59)_ = −3.0; *p* < .01**
Corsi block-tapping test span	**5.7**	**.9**	**5.3**	.9	37	2.7	29.7	*t*_(1, 65)_ = 1.7; *p* = .10
Sustained attention								
RVP (% of errors)^a^	**13.3**	8.8	**18.9**	11.3	37	13.5	35.1	***t*_(1, 65)_ = −2.2; *p* = .03**
*Note.* Percentage of phenylketonuria (PKU) participants with *z* score =>2 (expected % 2.3) is also reported. WCST = Wisconsin Card Sorting Test; RVP = Rapid Visual Information Processing.								
^a^ Tasks where there is a significant difference with controls.								

**Table 4 tbl4:** Speed and Accuracy in Orthographic and Spoken Language Tasks and Verbal Memory and Learning Tasks in Control and PKU Participants

	Controls		PKU participants					Controls vs. PKU
Domain/task	*M*	*SD*	*M*	*SD*	*N*	% *z* = > 2	% *z* => 1	*t* and *p*
Orthographic language								
Word reading								
RT − ms^a^	**507**	93.3	**574**	120	30	6.7	30	***t*_(1, 58)_ = −2.4; *p* = .02**
% errors	**.6**	.8	**.7**	1	30	10	16.7	*t*_(1, 58)_ = −.2; *p* = .84
Nonword reading								
RT − ms^a^	**604**	103.6	**795**	296.7	31	38.7	51.6	***t*_(1, 59)_ = −3.3; *p* < .00**
% errors	**5.8**	5.7	**7.9**	10.8	31	12.9	22.6	*t*_(1, 59)_ = −1.0; *p* = .33
Word spelling (% errors)	**4.8**	5.7	**3.9**	4.7	29	3.4	13.8	*t*_(1, 57)_ = .7; *p* = .50
Nonword spelling (% errors)	**11.9**	7.6	**13.3**	8.2	35	8.6	20	*t*_(1, 63)_ = −.7; *p* = .49
Spoonerisms (% errors)	**6.8**	6.6	**10.9**	14.7	30	13.3	23.3	*t*_(1, 58)_ = −1.4; *p* = .17
Phoneme deletion (% errors)	**11.2**	8.7	**14.5**	13.1	35	14.3	25.7	*t*_(1, 63)_ = −1.2; *p* = .24
Spoken language								
Pictures naming								
RT − ms	**835**	138.1	**897**	138.9	29	3.4	27.6	*t*_(1, 57)_ = −1.7; *p* = .09
% errors	**7.1**	5.1	**8.5**	11	30	3.3	6.7	*t*_(1, 58)_ = −.6; *p* = .53
Color naming								
RT − ms^a^	**617**	98.2	**731**	141.8	29	17.2	44.8	***t*_(1, 57)_ = −3.6; *p* < .01**
% errors	**.1**	.2	**.1**	.2	29	3.4	6.7	*t*_(1, 57)_ = −.1; *p* = .89
Vocabulary WASI^a^ (correct answer raw score)	**64.2**	7.2	**58.4**	8.7	37	10.8	40.5	***t*_(1, 65)_ = −2.9; *p* < .01**
Similarities WASI^a^ (correct answer raw score)	**39.2**	3.6	**36**	5.8	37	29.7	56.8	***t*_(1, 65)_ = −2.6; *p* = .01**
Verbal memory and learning								
Rey Auditory Verbal Learning Test								
Trial A1–A5 (% errors)	**20.5**	8.4	**24.1**	11.2	31	16.1	32.3	*t*_(1, 59)_ = −1.4; *p* = .17
Retention (% errors A6)	**15.8**	16.1	**18.3**	15.6	31	3.2	22.6	*t*_(1, 59)_ = −.6; *p* = .54
Delayed recall (% errors)	**15.1**	15.1	**16.3**	15.9	31	6.5	19.4	*t*_(1, 59)_ = −.3; *p* = .76
Paired Associate Verbal Learning								
Trial 1–5 (% errors)	**47**	24.9	**43.5**	*22.9*	31	0	12.9	*t*_(1, 59)_ = .6; *p* = .58
Delayed recall (% errors)	**29.3**	24.5	**20.4**	*23.7*	31	3.2	9.7	*t*_(1, 59)_ = 1.4; *p* = .16
*Note.* Percentage of phenylketonuria (PKU) participants with *z* score = > 2 (expected % 2.3) is reported. WASI = Wechsler Abbreviated Scale of Intelligence.								
^a^ Tasks where there is a significant difference with controls.								

## Footnotes

1 Note that a limit of the Complex Figure Test (Rey, 1941) when used to test visuospatial memory is that it also taps executive function skills because it requires planning and ongoing monitoring of performance (Watanabe et al., 2005). Thus, it is not clear whether an impairment is due to a visuospatial deficit or an executive deficit.
