# Supplementary material for: Cognitive Outcomes in Early-Treated Adults With Phenylketonuria (PKU): A Comprehensive Picture Across Domains
Source: Neuropsychology. 2017 Jan 12;31(3):255–67. doi: 10.1037/neu0000337 (PMC5328133; doi:10.1037/neu0000337)
Supplement: Supplementary file 1 [file z6o001172923so1.docx]

**Supplemental Materials**

**Cognitive Outcomes in Early-Treated Adults With Phenylketonuria (PKU): A Comprehensive Picture Across Domains**

**by L. Palermo et al., 2017, *Neuropsychology***

**http://dx.doi.org/10.1037/neu0000337**

**More detailed task description**

Visuo-spatial Attention

*Simple Detection.* Participants were asked to press a response button as soon as a ladybird appeared on the screen. The task included 20 trials. Presentation of the stimulus was preceded by a fixation cross displayed for 1000ms. The interval between the fixation point and presentation of the ladybird was variable (1-3 seconds). Performance was scored using the participant’s average speed of response.

*Detection with Distractors.* Participants had to respond to a ladybird as soon as it appeared on the screen by pressing a response key. As before, the stimulus appeared at a random interval after fixation (1-3 seconds) and was displayed until a response was made. Ladybirds were interspersed with green bugs and they could appear either alone or together or with a green bug. The participant had to press a button whenever the ladybird appeared on the screen alone or with a green bug. Failures to respond were counted as errors as were responses to the wrong stimulus (maximum number of errors 64). After the task was run once, it was repeated by reversing the required response (i.e., green bug; reversed target detection, N=64). Across the two parts of the task there were 128 trials overall, half with and half without the target.

*Choice Reaction Time* (from Cambridge Neuropsychological Test Automated Battery, CANTAB). In this task, an arrow-shaped stimulus appeared either the left or the right hand side of the computer screen. Participants were asked to press the button on the left of the response box, if the arrow pointed to the left and the button on the right, if the arrow pointed to the right. The side of presentation and the direction of pointing were always congruent with each other. The stimulus appeared at a random interval after fixation (1-750 ms) and was displayed for a maximum of 3100ms. If no response was made during this time, an error was recorded. Performance was scored in terms of number of errors and speed of response (maximum number of errors 100).

*Visual search.* Participants were asked to indicate if a red ladybird (target) was present among distractors (4, 8, or 12 items) by pressing a ʽyes’ or ʽno’ button. There were two conditions. In *Feature search*, distractors were green bugs, which differed from the target in both colour (green vs. red) and shape (bug vs. ladybird). In *Conjoined search*, there were also red bugs that differed from the target in shape, but not colour. A fixation cross was displayed for 1 second and the stimuli were presented immediately afterwards. They were displayed until a response was made. Each condition, simple and conjoined, included 36 trials.

Visuo-spatial Memory and Learning

*Delayed Matching to Sample* (DMS; adapted from Sahakian et al., 1988). This task assesses forced choice recognition memory for visual patterns. Participants were shown a complex visual pattern (target) on a computer screen which they had to match with one of four similar patterns (the target plus three distractors). The target and the choice patterns were shown simultaneously or after a delay of 0, 4 or 12 seconds. The task included 10 trials for each condition (maximum number of errors 40).

*Paired Associate Visual Learning* (PAL; adapted from Sahakian et al., 1988). The purpose of this task is learning to associate objects with locations. Participants were presented with a number of white boxes (6 or 8) which opened up in a random order, one at a time, to show 3, 6 or 8 visual patterns. Participants had to remember which pattern belonged to which box. After the last box opened, the patterns were displayed in the middle of the screen, one at a time, and the participants had to touch the box where the pattern was originally located. If participants made an error, the patterns were re-presented to remind the participant of their locations (maximum 10 attempts per trial). There were 2 trials with 3 patterns (N=60 patterns), 1 with 6 patterns (N=60 patterns), and 1 with 8 patterns (N=80 patterns; maximum number of errors 200).

Visuo-motor Coordination

*Grooved Pegboard Test* (Trites, 1977). This test requires both visual-motor coordination and fine motor control skills. The apparatus consisted of a pegboard with 25 holes and 25 pegs. The holes have grooves and the pegs have to be appropriately rotated to be inserted in the holes. The participants had to fill the board with pegs as quickly as possible using only one hand. Two trials each were carried out with the dominant and non-dominant hand. Performance was scored as the number of seconds required to complete the task.

*Digit symbol-coding* (Wechsler, 1997). Participants were given a key grid of numbers and matching symbols and a test section with a series of numbers and empty boxes below them. The task consisted of filling in as many boxes as possible with the corresponding symbols in 90 seconds. Each incorrectly matched number–symbol and each empty box counted as an error (maximum number of errors= 93).

Complex executive functions

*The Wisconsin Card Sorting Test – 64 Card Version* (WCST-64; Kongs, Thompson, Iverson, & Heaton, 2000) is an abbreviated form of the standard 128-card version. This test assesses the ability to derive rules and use feedback to shift cognitive set. Participants were presented with four place-holding cards depicting symbols differing in colour (green, red, blue, and yellow), number (1-4), and shape (circles, triangles, crosses and stars). Participants were then given a deck of 64 cards and asked to match each card with the corresponding place-holding card. Participants were not told what stimulus dimension (colour, number or shape) to use to match the cards, but feedback was provided after each choice. Once the participant made 10 consecutive correct matches for a predetermined sorting category (e.g., colour), the sorting rule was changed (e.g., shape) without telling the participant and he/she had to discover the new sorting rule.

*Trail Making Test B-A* (AITB, 1944; Sánchez-Cubillo et al., 2009)*.* This task requires drawing a trail with a pencil on a piece of paper by joining a number of circles. In version A of the task, the circles only contained numbers. In version B, they contained either numbers (1 – 13) or letters (A – L). Participants had to connect the circles in ascending order as quickly as possible. In version B, they had to alternate between the numbers and the letters (i.e., 1-A-2-B, etc.). Performance was scored in terms of the number of seconds required to complete the task. The difference between version A and B taps executive functions, since Brequires planning and shifting of criteria.

*Tower of Hanoi puzzle* (ToH, Shallice, 1982) assesses the ability to plan ahead to solve a problem. Participants were asked to move a tower of rings (i.e., 3, 4 or 5 rings) of different sizes staked on one peg, in decreasing size order, on a different peg, passing through an intermediate peg and following a set of rules (i.e., a larger ring cannot be placed on top of a smaller ring and only one ring can be moved at a time). Problems of increasing complexity were presented, defined by the smallest number of moves necessary to solve the problem (from a 7-move to a 31-move problem). A trial was ended if, after 6 minutes, the participant was not able to reach the goal configuration. Since many PKU participants were not able to solve the trials, performance was scored in terms of total number of unresolved trials and not in terms of the number of movements necessary to solve each trial (max number of unsolved trials 9). Tower tests do not appear to have been used in previous AwPKU assessments. In children results are inconsistent: impaired performance was reported by Welsh, Pennington, Ozonoff, Rouse, and McCabe (1990) but not by Anderson, Anderson, Northam, Jacobs, and Mikiewiez (2002), Stemerdink et al. (1999) and VanZutphen et al. (2007).

*Verbal Fluency* This test measures lexical access, but also strategic search through the lexicon. In the *Letter Fluency* condition (Benton, Hamsher, & Sivan, 1994), participants were required to produce as many words as possible beginning with a given letter in a minute of time. The letters ʽcʽ, ʽfʽ and ʽl’ were used. Participants were asked not to use proper nouns or words sharing the same root. In the *Semantic Fluency* condition (Rosen, 1980), participants were required to generate as many words as possible belonging to a specific category (animals) in 1 min of time. For both subtests scoring was based on the number of acceptable words produced.

Inhibitory Control

*The Stroop Colour-Word Test* (Stroop, 1935). The Stroop test evaluates the ability to inhibit inappropriate responses following an explicit task instruction. Participants were asked to report the ink colour of three types of stimuli shown on the computer screen: a sequence of ʽX’ letters (ʽXXXX’, *neutral condition*), coloured words where the colour of the ink matched the meaning of the word (“red” written with red ink; *congruent condition*), coloured words where the colour of the ink was incongruent with the meaning of the word (e.g. ʽred’ written with yellow ink; *incongruent condition*). To succeed in the incongruent condition, participants had to suppress the tendency to *read* the word. There were 24 trials for each condition. Reaction times (RTs) were recorded via a voice-key. The neutral condition served as a measure of ability to name the colours. The interference effect was measured as the difference between the incongruent and congruent condition.

*Semantic Interference*. This was measured as the increase in naming latencies between the first and last exemplar of a series of semantically related nouns (see Picture naming later on). Semantic interference arises when activation of competing representations cannot be properly inhibited.

Short-term memory/working memory

*Digit span.* Participants were asked to repeat a sequence of digits spoken by the examiner at the rate of approximately one per second. The sequences ranged from four to eight digits (*N* = 10 for each length). The task ended when the participants could not recall more than half of the sequences of a certain length. To calculate the span, a value of 0.1 was assigned to each sequence repeated correctly (maximum span 8). Each length was scored one point if all the sequences were correct and 3 points were added as a baseline.

*Nonword repetition (*Romani, Tsouknida & Olson, 2015). Participants were asked to repeat a sequence of nonwords spoken by the examiner. There were 10 sequences of 2 nonwords, 10 of 3 nonwords, and 10 of 4 nonwords. Nonwords respected the phonotactic constraints of the English language. The task ended when less than half of the sequences of a given length were repeated correctly. One error was counted for each sequence repeated incorrectly (maximum number of errors, 30).

*Corsi Block Span* (Corsi, 1972). The examiner tapped a sequence of blocks at the rate of one per second, and immediately afterwards participants attempted to reproduce the sequence in the same order. Sequences of increasing length (from 1 to 9) were presented with 3 trials for each length. The task was stopped when the participant failed to reproduce all three sequences of a given length. For each length, one point was given if all three sequences were reproduced correctly, 0.66 if two were correct and 0.33 points if only one sequence was correct.

Sustained Attention

*Rapid Visual Information Processing task* (RVP; adapted from Sahakian, Jones, Levy, Gray, & Warburton, 1989). This task assesses the ability to maintain attention over time. Digits ranging from 2 to 9 appeared on the screen, one at a time, at a rate of 100 digits per minute. Participants had to detect three target sequences of 3 digits (i.e., 2-4-6, 3-5-7, 4-6-8) by pressing the response key when the last number of the sequence appeared on the screen STM component was minimized by having the three target sequences (i.e., 2-4-6, 3-5-7 and 4-6-8) written on the screen as a reminder for the participant. There were four blocks of 19 trials each (76 trials in total). Each trial contained a variable number of digits (from 3 to 10). For each block, 9 trials contained the sequence and 10 did not. An error was scored for each incorrect answer (e.g. pressing the response key at the wrong time) or omission (i.e. not pressing the response key for the last number of a sequence) within a window including the following 2 digits (1800 ms; maximum number of errors 76).

Orthographic Language

*Word/non-word spelling (*Romani, Tsouknida & Olson, 2015)*.* Stimuli were presented spoken, one at a time, for spelling to dictation with no time limit for the response. In case of self-corrections, the last response was scored. Homophones were presented with a disambiguating sentence. Non-words were obtained by substituting one or two phonemes in real English words. 140 regular and irregular words of various frequencies and lengths and 40 non-words were used. They were presented blocked. For non-words, all phonologically plausible renditions of the items were accepted as correct (for instance, both HUSCANT and HUSKANT were correct alternatives for HUSCANT, pronounced like “husband”). In case of self-corrections, only the last response was scored (maximum number of errors: for words=140; non-words=40).

*Word/non-word reading* (adapted from Romani, Tsouknida & Olson, 2015)*.* Participants were asked to read aloud 140 regular and irregular words. Words appeared one at a time at the centre of a computer screen. Each word was preceded by a fixation cross for 1000 ms and disappeared 500 ms after a response was made. Reaction times (RTs) were recorded via a voice-key (maximum number errors, 140). The same procedure was used with 40 non-words that were obtained by changing one or two letters in a similar set of words. Reaction times (RTs) were recorded via a voice-key (maximum number errors, 40).

Among orthographic tasks we also included spoonerisms and phoneme deletion tasks. These are tasks which rely heavily on knowledge of orthographic representations and are impaired in developmental dyslexia (Landerl, Wimmer, & Frith, 1997; Romani, Tsouknida & Olson, 2015).

*Spoonerisms (*adapted from/ a short version of Romani, Tsouknida & Olson, 2015)*.* Participants heard two spoken words (e.g. bad-sin; dare-night; 24 pairs) and were asked to exchange the initial sounds to produce either two different words (bad-sin > sad-bin) or two non-words (dare-night > nare-dight). There were no time limits to respond (maximum number of errors, 48).

*Phoneme deletion.* Participants heard a spoken English word (e.g., table) and then one sound (/t/). They were asked to repeat back the word, but without the sound (e.g., able). Taking away the sound resulted in a real word half of the time and in a non-word the other half (e.g., powder; /d/ >power; cabbage; /k/ > abbage). The task included 40 stimuli (maximum number of errors, 40).

Spoken Language

*Picture naming.* We used a task similar to that originally used by Howard, Nickels, Coltheart, and Cole-Virtue (2006), which allows us to measure accuracy and speed in lexical access, but also an effect of semantic interference that arises when pictures belonging to the same semantic category are named in succession, one after the other. 165 pictures were presented, one at a time, on a computer screen. Participants were instructed to say the name as fast as possible. All pictures were black and white line drawings of common objects. 120 pictures belonged to 24 different semantic categories (5 items in each category) and the remaining 45 were fillers. They were presented in a randomized order. The number of pictures between successive members of the same category varied from two to eight. Reaction times (RTs) were recorded via a voice-key. Previous results have shown that RTs progressively increase with the ordinal position in the set of semantically related pictures (Howard et al., 2006; Oppenheim, Dell, & Schwartz, 2007). We used overall RT average and rate of errors as measures of lexical access. We measured the semantic interference effect as the increase in RT and errors from the first to the last picture in a related set. This was considered a measure of (lack of) inhibitory control (see above).

*Color naming.* Here we included the neutral (XXX) and congruent conditions of the Stroop task where participants have to name, as fast as possible, the colour of the stimulus appearing on the screen.

*Vocabulary* (WASI; Wechsler, 1999). This task measures word knowledge, expressive verbal skills and verbal concept formation. Participants were asked to define up to 42 English words presented both verbally and written. Answers to items 1-4 were scored 0 or 1; answers to items 5-42 were scored 0, 1, or 2 points according to the quality of the answer (maximum raw score 80).

*Similarities* (WASI; Wechsler, 1999). This task measures verbal concept formation and verbal reasoning. The participants were presented with up to 26 pairs of words. They were asked to describe how similar the two words in the pair were. Answers to items 1-4 were scored 0 or 1; answers to items 5-26 were scored 0, 1, or 2 points according to the quality of the answer (maximum raw score 48).

Verbal Memory and Learning

*The Rey Auditory Verbal Learning Test* (RAVLT; Rey, 1964; Schmidt, 1996). This test evaluates the ability to encode, consolidate and retrieve verbal information. We used the 'standard' presentation. Participants were first presented with a list of 15 nouns (List A) and asked to recall as many words as they could in any order immediately after presentation. List A was repeated five times to assess learning. After that, a second list (List B) was presented to assess interference. Recall of list A was assessed again immediately after recall of the interfering list B and after a 20 minute delay.

*Paired associates verbal learning (*Romani, Tsouknida & Olson, 2015)*.* Here, participants have to learn the association between a made-up word and the picture of an object or animal (similar to learning foreign vocabulary). In an initial phase, participants were presented with nine pictures, each associated with a written novel word, and were asked to copy the word down. After that, they were asked to write the correct novel word on presentation of the picture alone (testing phase). In case of errors, the correct word was presented and the participants were asked to copy it down. The task was discontinued when all the words in the list were recalled correctly or after a maximum of five attempts at the whole list. When testing was discontinued after a completely correct list, all subsequent words were counted as correct (maximum number of errors, 45). After 20 minutes (delayed recall) participants were presented again with the nine pictures and asked to write the correct associated words (maximum number of errors, 9).

**References for more detailed task description**

Anderson, V. A., Anderson, P., Northam, E., Jacobs, R., & Mikiewiez, O. (2002). Relationship between cognitive and behavioral measure of executive function in children with brain disease. *Child Neuropsychology, 8*, 231–240.Baddeley, A. (1986). *Working memory*. Oxford, England: Clarendon Press.

Benton, A. L., Hamsher, K. de S., & Sivan, A. B. (1994). *Multilingual Aphasia Examination*. Iowa City: AJA Associates.

Corsi, P. M. (1972). Human memory and the medial temporal region of the brain. McGill University, Montreal: Unpublished doctoral dissertation.

Howard, D., Nickels, L., Coltheart, M., & Cole-Virtue, J. (2006). Cumulative semantic inhibition in picture naming: experimental and computational studies. *Cognition, 100*, 464–482.

Kongs, S.K. , Thompson , L.L. , Iverson , G.L. , & Heaton , R.K. ( 2000 ). *WCST-64: Wisconsin Card Sorting Test-64 card version, professional manual.* Odessa, FL: Psychological Assessment Resources.

Landerl, K., Wimmer, H., & Frith, U. (1997). The impact of orthographic consistency on dyslexia: a German-English comparison. Cognition, *63*, 315-334.

Oppenheim, G.M., Dell, G.S., Schwartz, M.F. (2007). Cumulative semantic interference as learning. *Brain and Language, 103*, 175–176.

Rey, A. (1964). *L’examen clinique en psychologie* [Clinical examination in psychology]. Paris: Presses Universitaires de France.

Romani, C., Tsouknida, E., & Olson, A. (2015). Encoding order and developmental dyslexia: A family of skills predicting different orthographic components. *Quarterly Journal of Experimental Psychology, 68*, 99–128.

Rosen, W.G. (1980). Verbal fluency in aging and dementia. *Journal of Clinical Neuropsychology, 2*, 135–46.

Sahakian, B. J., Jones, G. M. M., Levy, R., Gray, J. A., & Warburton, D. M. (1989). The effects of nicotine on attention, information processing, and short-term memory in patients with dementia of the Alzheimer type. *British Journal of Psychiatry, 154*, 797-800.

Sahakian, B. J., Morris, R.G., Evenden, J.L., Heald, A., Levy, R., Philpot, M., & Robbins, T.W. (1988). A Comparative Study of Visuospatial Memory and Learning in Alzheimer-Type Dementia and Parkinson's Disease. *Brain, 111*, 695–718.

Sánchez-Cubillo, I., Periáñez, ,J.A., Adrover-Roig, D., Rodríguez-Sánchez, ,J.M., Ríos-Lago, M., Tirapu, J., & Barceló, F. (2009). Construct validity of the trail making test: Role of task-switching, working memory, inhibition/interference control, and visuomotor abilities. Journal of the International Neuropsychological Society, 15, 438-50.

Schmidt, M. (1996). *Rey auditory verbal learning test: A handbook*. Los Angeles, CA: Western Psychological Services.

Shallice, T. (1982). Specific impairments of planning. *Philosophical transactions of the Royal Society of London, 298,* 199-209.

Stemerdink, N., Van Der Molen, M., Kalverboer, A., Van Der Meere, J., Huisman, J., De Jong, L., … Van Spronsen, F. (1999). Prefrontal dysfunction in early and continuously treated phenylketonuria, *Developmental Neuropsychology, 16*, 29–57.

Stroop, J. R. (1935). Studies of interference in serial verbal reactions. *Journal of Experimental Psychology, 18,* 643–662.

Trites, R. (1977). *Grooved Pegboard Test*. Lafayette, IN: Lafayette Instrument.

VanZutphen, K., Packman, W., Sporri, L., Needham, M., Morgan, C., Weisiger, K., & Packman , B. (2007). Executive functioning in children and adolescents with phenylketonuria. *Clinical genetics,* *72*, 13-18.

Wechsler, D. (1991). *The Wechsler intelligence scale for children—third edition (WISC-III).* San Antonio, TX: The Psychological Corporation.

Wechsler, D. (1997). Wechsler Adult Intelligence Scale—Third Edition. San Antonio, TX: The Psychological Corporation.

Wechsler, D. (1999). *Wechsler Abbreviated Scale of Intelligence (WASI).* San Antonio, TX: Harcourt Assessment.

Welsh, M., Pennington, B., Ozonoff, S., Rouse, B., & McCabe, E. (1990). Neuropsychology of early-treated phenylketonuria: specific executive function deficits. *Child Development, 61,* 1697–1713.
